# The Role of Pattern Extrapolation in the Perception of Dynamic Facial Expressions in Autism Spectrum Disorder

**DOI:** 10.3389/fpsyg.2018.01918

**Published:** 2018-10-15

**Authors:** Letizia Palumbo, Sylwia T. Macinska, Tjeerd Jellema

**Affiliations:** ^1^Department of Psychology, Liverpool Hope University, Liverpool, United Kingdom; ^2^Psychology, School of Life Sciences, University of Hull, Hull, United Kingdom

**Keywords:** dynamic facial expressions, perceptual distortions, pattern extrapolation, emotional anticipation, embodied simulation

## Abstract

Changes in the intensity and type of facial expressions reflect alterations in the emotional state of the agent. Such “direct” access to the other’s affective state might, top-down, influence the perception of the facial expressions that gave rise to the affective state inference. Previously, we described a perceptual bias occurring when the last, neutral, expression of offsets of facial expressions (joy-to-neutral and anger-to-neutral), was evaluated. Individuals with high-functioning autism (HFA) and matched typically developed (TD) individuals rated the neutral expression at the end of the joy-offset videos as slightly angry and the identical neutral expression at the end of the anger-offset videos as slightly happy (“overshoot” bias). That study suggested that the perceptual overshoot response bias in the TD group could be best explained by top-down “emotional anticipation,” i.e., the involuntary/automatic anticipation of the agent’s next emotional state of mind, generated by the immediately preceding perceptual history (low-level mind reading). The experimental manipulations further indicated that in the HFA group the “overshoot” was better explained by contrast effects between the first and last facial expressions, both presented for a relatively long period of 400 ms. However, in principle, there is another, more parsimonious, explanation, which is pattern extrapolation or representational momentum (RM): the extrapolation of a pattern present in the dynamic sequence. This hypothesis is tested in the current study, in which 18 individuals with HFA and a matched control group took part. In a base-line condition, joy-offset and anger-offset video-clips were presented. In the new experimental condition, the clips were modified so as to create an offset-onset-offset pattern within each sequence (joy-to-anger-to-neutral and anger-to-joy-to-neutral). The final neutral expressions had to be evaluated. The overshoot bias was confirmed in the base-line condition for both TD and HFA groups, while the experimental manipulation removed the bias in both groups. This outcome ruled out pattern extrapolation or RM as explanation for the perceptual “overshoot” bias in the HFA group and suggested a role for facial contrast effects in HFA. This is compatible with the view that ASD individuals tend to lack the spontaneous “tracking” of changes in the others’ affective state and hence show no or reduced emotional anticipation.

## Introduction

The dynamic expressivity of the face greatly facilitates social communication. Very subtle changes in facial expressivity can be detected and may reflect subtle positive or negative alterations in the affective state of the agent (Krumhuber et al., 2013). The ability to detect such emotional state alterations over time enables us to make predictions about other people’s behavior. Typically, we read facial expressions without explicit intention to do so or without inferential efforts. This ability to tacitly understand others’ mental states has been referred to as low-level mind reading (Goldman, 2006). Its implicit (automatic, non-volitional) nature can be contrasted to the deliberate, effortful, use of cognitive resources or conceptual and linguistic mediators, involved in explicit Theory of Mind (Baron-Cohen, 1995), which is referred to by Goldman as high-level mind reading (Goldman, 2006).

There is growing evidence that these implicit, involuntary or spontaneous, skills for reading others’ emotional or mental states are compromised in Autism Spectrum Disorder (ASD; Hudson et al., 2009, 2012; Jellema et al., 2009; Senju et al., 2009), and possibly also in other disorders like schizophrenia (Van ’t Wout et al., 2009) and OCD (Obsessive compulsive disorder; Tumkaya et al., 2014), resulting in inadequate social exchanges. ASD is a pervasive neurodevelopment condition characterized by impaired social development and stereotypical, repetitive behaviors, often associated with obsessive interests and a lack of empathy (Rutter, 1978; World Health Organization [WHO], 2008; DSM-V, 2013). Symptom severity varies hugely in ASD (DSM-V, 2013). High-functioning autism (HFA) is a relatively mild form of ASD with normal intelligence quotient (IQ) distribution, but with a delayed development of language skills and difficulties in social and emotional domains (McPartland and Klin, 2006).

The perceptual processing stage of social cues, such as facial expressions, gaze direction, bodily postures, and action sequences, provides a mechanistic description of these cues, grounded on physical or geometrical features and dynamics of the stimuli, possibly in relation to contextual cues and objects in the environment (Jellema and Perrett, 2003, 2006, 2012). These mechanistic descriptions next trigger inferences about the emotional/mental state of the agent (e.g., Blakemore and Decety, 2001). Besides this bottom-up route (from perception of bodily cues to attribution of social meaning), there is also a top-down route where attributions of other people’s mental states (such as intentions) can in turn influence the low-level perception of bodily cues (Hudson et al., 2009; Teufel et al., 2009). These top-down processes can be highly inferential or reflective, but they can also be quite reflexive and automatic (Satpute and Lieberman, 2006; Lieberman, 2007). The bi-directional interaction between bottom-up and top-down streams has been captured under the term “perceptual mentalizing” (Teufel et al., 2010). This model, however, only considers explicit mental attributions, neglecting the possibility that implicit attributions might also influence social perception. Hudson et al. (2009) reported a study where participants’ estimations of how far an agent’s head had rotated were influenced by the agent’s gaze direction. With gaze direction ahead of head rotation the head rotation was overestimated as compared to when the gaze was lagging behind head rotation. Importantly, participants were not aware of the eye gaze manipulation. The bias thus seemed to be induced by implicit attributions of the intention to continue/discontinue to move in the direction of the head rotation. This study therefore supports the idea that early stages of the visual processing of social stimuli can be influenced by implicit attributions made by the observer about the agent’s mental state.

In individuals with ASD an impaired top-down route would result in relatively unbiased perception of bodily cues, not “contaminated” by attributions of mental/emotional states (cf. Wang and Hamilton, 2012). However, at the same time it might make them more susceptible to perceptual illusions driven by low-level perceptual features, such as geometries or patterns present in the stimuli. In this respect, it is interesting to note that the perceptual bias reported in TD individuals in Hudson et al. (2009) was also found in individuals with ASD (Hudson and Jellema, 2011). However, in contrast to the TD group, the ASD group continued to show this bias in response to a non-social stimulus designed to match the low-level physical characteristics (eye gaze) of the agent stimulus. These results suggest that individuals with ASD fail to grasp the mental states in an involuntary manner and instead rely on the mechanistic descriptions of the physical features of the social cues (such as directional cues, input-output relations, or statistical regularities).

### Emotional Anticipation in TD and HFA

In a series of studies, we presented a phenomenon occurring when evaluations have to be made of dynamic offsets of facial expressions (Jellema et al., 2011; Palumbo and Jellema, 2013; Palumbo et al., 2015). TD participants observed dynamic presentations of an intense facial expression of joy or anger, which gradually weakened until the actor posed a neutral expression. Participants’ task was to evaluate the last neutral frame on a 5-point Likert scale, ranging from slightly angry (1), via neutral (3), to slightly happy (5). Results showed a perceptual bias (which we call “overshoot” bias), such that the neutral expression at the end of the joy-to-neutral videos was evaluated as slightly angry, and the identical neutral expression at the end of anger-to-neutral videos as slightly happy (in the remainder of the text we refer to this condition as the Offset condition). We proposed that the perceptual history led the observer to automatically anticipate what the emotional state of the agent would be *after* the sequence stopped. The “emotional anticipation” is thought to drive, in a top-down fashion, the perceptual bias. This interpretation fits with the “perceptual mentalizing” model proposed by Teufel et al. (2010). However, as emotional anticipation acted involuntarily’, it highlights the role of implicit attributions in social perception.

In subsequent studies, we found that participants with HFA also reported the perceptual overshoot bias (Palumbo et al., 2015). However, when in an additional condition we changed the identity of the agent in the last frame of the video-clips (the new identity was unfamiliar to the observer), the influence of the perceptual history was nullified in the TD group (the overshoot bias was removed), while the HFA group continued to report an overshoot bias. This suggested that the perceptual distortion found in the TD group was not due to sequential contrast effects (Tanaka-Matsumi et al., 1995) as the degree of expressive contrast between the first and last frames of the videos remained unaffected by the identity change manipulation. The removal of the overshoot bias was compatible with the emotional anticipation hypothesis. Actor B in the last frame was someone for whom no perceptual history was available, so the observer did not know anything about B’s emotional state other than that B had a neutral expression, and therefore rated B as neutral. The finding that the HFA group continued to show an overshoot bias suggested that they had not used an anticipation mechanism linked to the agent (we established that they did detect the change in identity). We hypothesized that the persistence of the overshoot bias in the HFA group might have resulted from susceptibility to low-level stimulus features, most probably the contrast between the first (happy or angry) and the last (neutral) expression (both presented for a relatively long period of 400 ms), which is not affected by the identity change (Palumbo et al., 2015).

For the TD group, the results of the identity-change condition also seemed to rule out an explanation in terms of representational momentum (RM; Freyd and Finke, 1984; Yoshikawa and Sato, 2008), or at least suggest that in TD individuals RM can be modulated, or even overruled, by top-down information (such as information referring to the agent’s identity). RM is the phenomenon that an observer’s memory for the final position of a moving target is displaced further along the observed trajectory (Freyd and Finke, 1984), which also applies to the gradual changes in dynamic facial expressions (Yoshikawa and Sato, 2008). However, also for the HFA group, it is in principle possible that RM, rather than sequential contrast effects, could explain the response bias, as they continued to report the bias in the identity-change condition. These experiments therefore did not allow to discriminate between these two competing low-level explanations in the HFA group. Another condition (Palumbo et al., 2015) in which video-clips started with a neutral expression that morphed via happy (or angry) back to neutral (forming a “loop”) did not produce a bias in the evaluations of the HFA group. However, as the extrapolation direction in this condition is ambiguous (in which direction does the pattern continue?), it cannot be used to exclude RM as the underpinning mechanism.

Representational momentum at work in the base-line condition in HFA individuals would mean that the negative going trend (happy offset) would be extrapolated into a slightly angry expression, and that the positive going trend (angry-offset) would be extrapolated into a slightly happy expression. Individuals with HFA tend to be adept at detecting regularities, input-output relations or statistical regularities, which typically govern the physical world (Baron-Cohen, 2002; Baron-Cohen et al., 2003). In individuals with HFA, this tendency for low-level pattern detection and extrapolation may be quite prominent and may not easily get overruled by top-down information relating to the object, such as information that the agent’s identity had changed (cf. Vivanti et al., 2011).

### The Current Study

The current study aimed to clarify what drove the perceptual bias in the HFA group in our previous experiments, specifically targeting the role of extrapolation (or RM) of patterns present in the dynamic facial expressions. To this end video-clips were created in which an intense facial expression (happy or angry) gradually morphed via a neutral expression into its “opposite” expression (angry or happy), after which it morphed back to neutral (joy-to-anger-to-neutral and anger-to-joy-to-neutral sequences). The final neutral expressions of the videos were again evaluated using the 5-point Likert scale. The rationale was that if the overshoot effect is driven by pattern extrapolation (or RM) then the last, neutral, expression should be evaluated as slightly happy in the joy-to-anger-to-neutral videos, and as slightly angry in the anger-to-joy-to-neutral videos. In other words, observers would implicitly expect the pattern to continue. Pattern extrapolation and RM predict the same outcome in this paradigm: a slightly happy overshoot for the joy-to-anger-to-neutral videos, and a slightly angry overshoot for the anger-to-joy-to-neutral videos. Further, if the evaluations in the TD group would be driven by emotional anticipation (as suggested by Palumbo and Jellema, 2013), then we would predict the absence of a response bias in this new condition in the TD group, as in terms of “tracking and anticipating” the agent’s emotional state of mind, the videos would, if anything, suggest the agents to remain emotionally neutral after the clip stopped.

## Materials and Methods

### Participants

#### HFA Group

Twenty-one individuals with HFA participated in the experiment. All were recruited through disability services from universities in the North-East of England (United Kingdom).

They all had previously received a diagnosis of HFA or Asperger’s syndrome from a clinical psychologist or psychiatrist based on DSM-IV-TR (American Psychiatric Association [APA], 2013) or ICD-10 (World Health Organization [WHO], 2008) criteria. Diagnosis of HFA was confirmed using the ADOS (Autism Diagnostic Observation Schedule, module 4), administered by a qualified experimenter (SM). The ADOS is a semi-structured, standardized assessment of communication, social interaction, and imagination, designed for use with children and adults suspected of having ASD. They also completed the Autism Spectrum Quotient questionnaire (AQ; Baron-Cohen et al., 2001), which is a fifty-statement, self-administered questionnaire, designed to measure the degree to which an adult with normal intelligence possesses autistic-like traits. IQ scores were determined using the Wechsler Adult Intelligence Scale, WAIS-III (Wechsler, 1997). From the 21 students with HFA participating in the study, three were removed following the application of exclusion criteria to the data set (see Data reduction below for details). The remaining 18 students (6 females, 12 males; mean age = 19.9 years, SD = 1.1) had a mean total ADOS score of 9.3 (SD = 2.6) and a mean AQ score of 32.3 (SD = 9.5). Their mean total IQ score was 117.7 (SD = 8.1).

#### TD Group

All TD participants were undergraduate Psychology students from Hull University. All were asked if they had previously obtained a head injury or had received a diagnosis of ASD or another mental health or developmental disorder. No participants disclosed this. Twenty-two TD individuals took part in the study; applying the exclusion criteria to the data set (see below) removed four individuals. The remaining 18 participants (5 females, 13 males; mean age = 20.5 years, SD = 1.4) had a mean AQ score of 17.6 (SD = 3.9) and a mean total IQ score of 114 (SD = 6.7). The TD group did not differ from the HFA group in terms of age [*t*(34) = 1.43, *p* = 0.163], gender ratio [*X*^2^(1,35) = 0.13, *p* = 0.72], or IQ [*t*(34) = −1.56, *p* = 0.13]. As expected, AQ scores were significantly higher in the HFA group [*t*(34) = 7.59, *p* < 0.001]. Importantly, the HFA group matched very closely to the control TD group, as both groups consisted of university students with fairly similar daily routines, resulting in a good approximation of the influence of the factor “HFA.” All HFA and TD participants had normal or corrected-to-normal vision, and provided written informed consent prior to the experiment. Participants received course credits or a fee for taking part. The study was approved by the Ethics committee of the Department of Psychology of Hull University.

### Stimuli

**FIGURE 1:**
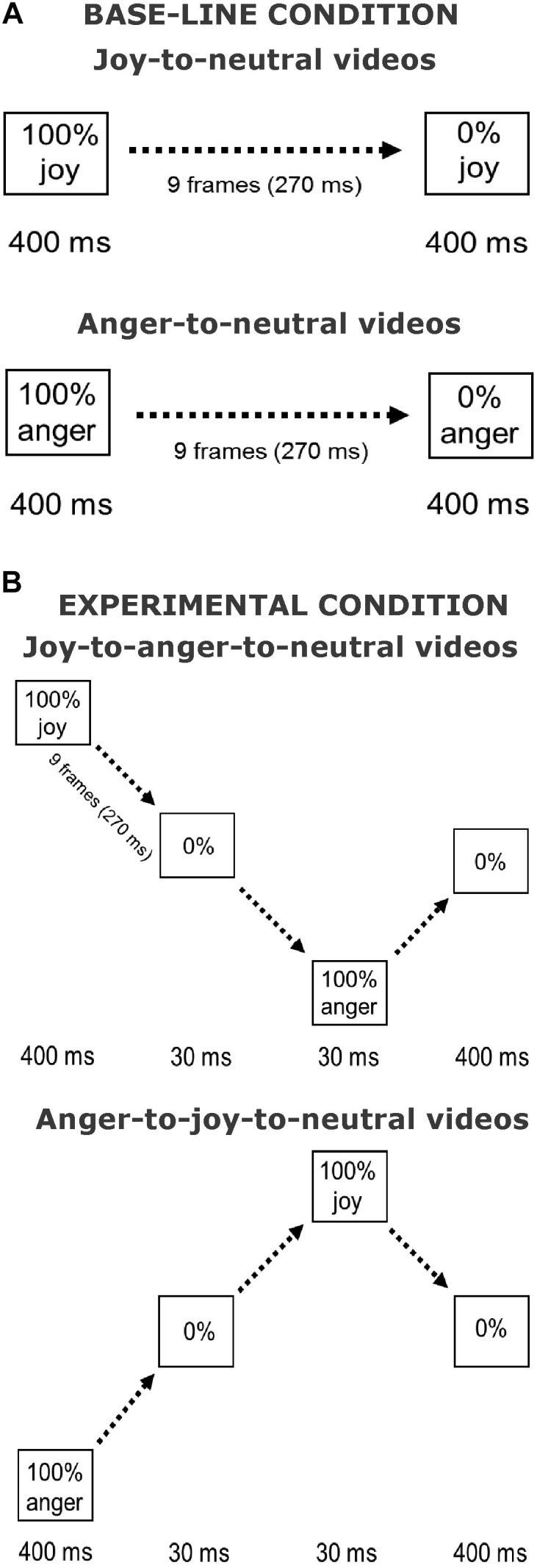
Illustration of stimulus presentations. Shown are the Offset condition **(A)** and the Offset-onset-offset condition **(B)**, for joy and anger initial emotions. Face pictures are shown in Palumbo and Jellema (2013).

The stimuli used in the current experiment were similar to those used in Palumbo and Jellema (2013) and in Palumbo et al. (2015). Pictures of facial expressions of joy and anger were selected from the Pictures of Facial Affect (eight actors, four males: EM, JJ, PE, WF, and four females: C, MO, PF, SW) (Ekman and Friesen, 1976; Young et al., 2002). All faces were frontally oriented with their eye gaze directed straight ahead. The photographs were in grayscale. The pictures were digitally adjusted to match in contrast and brightness. The eyes of all actors were positioned at approximately the same screen location. Faces measured about 13 × 20 cm when displayed on the screen, subtending approximately 8° vertically. Nine interpolated images, in between the full-blown expression of joy or anger (which is called 100%) and the neutral expression (0%) were created at equal steps of 10% intensity change, using computer morphing procedures (Perrett et al., 1994). In the Offset condition, the morph sequences depicted a maximally happy or angry expression of which the intensity gradually decreased until a neutral expression was reached (joy-to-neutral or anger-to-neutral). The first and last frames of the sequences were displayed for 400 ms. The duration of the morph sequence was 270 ms (9 frames × 30 ms), the total duration of the stimulus presentation was 1070 ms. In the new condition, the initial full-blown facial expression (happy or angry) gradually morphed smoothly via a neutral expression into its “opposite” expression, after which it morphed back to neutral (joy-to-anger-to-neutral and anger-to-joy-to-neutral sequences). We will refer to this manipulation as the offset-onset-offset condition. The first and the last frames again both lasted 400 ms. The duration of these morph sequences was 870 ms (29 frames × 30 ms), the total duration of the stimulus presentation was 1940 ms. An illustration of the morph sequences in both conditions is presented in **Figure 1**.

### Experimental Procedure

Participants were seated at a viewing distance of 80 cm from a PC screen (17-inch monitor, 1024 × 768 pixels, 100 Hz). The stimuli were presented using E-Prime (v. 1.2; Psychology Software Tools, Inc.). The software uploaded each single frame at specific durations as illustrated in **Figure 1**. This generated smooth morph sequences that resembled short video clips. First participants completed a calibration phase in which they rated the static neutral expressions of the eight actors (i.e., neutral expressions according to the ratings from Ekman and Friesen, 1976). Each calibration trial started with a fixation cross displayed in the center of the screen for 500 ms, followed by the static neutral face displayed for 600 ms. Sixteen calibration trials were presented (eight actors, two repetitions each) in randomized order. Participants were prompted to rate these “neutral” expressions using a 5-point scale, ranging from slightly angry (1) via neutral (3) to slightly happy (5), by pressing one of the five labeled keys on a button box (SR-Box, Psychology Software Tools, Inc., United States). Directly following the calibration phase, the experimental session started. First, 6 practice trials were completed (displaying two actors not used in the experiment), followed by 64 randomized experimental trials (8 actors × 2 expressions × 2 conditions × 2 repetitions). Each trial started with a fixation cross displayed for 500 ms, followed by the video-clips. Participants were prompted to rate the last neutral expression of the sequence using the same 5-point scale, and were instructed to respond within 3 s.

## Results

### Calibration

**FIGURE 2:**
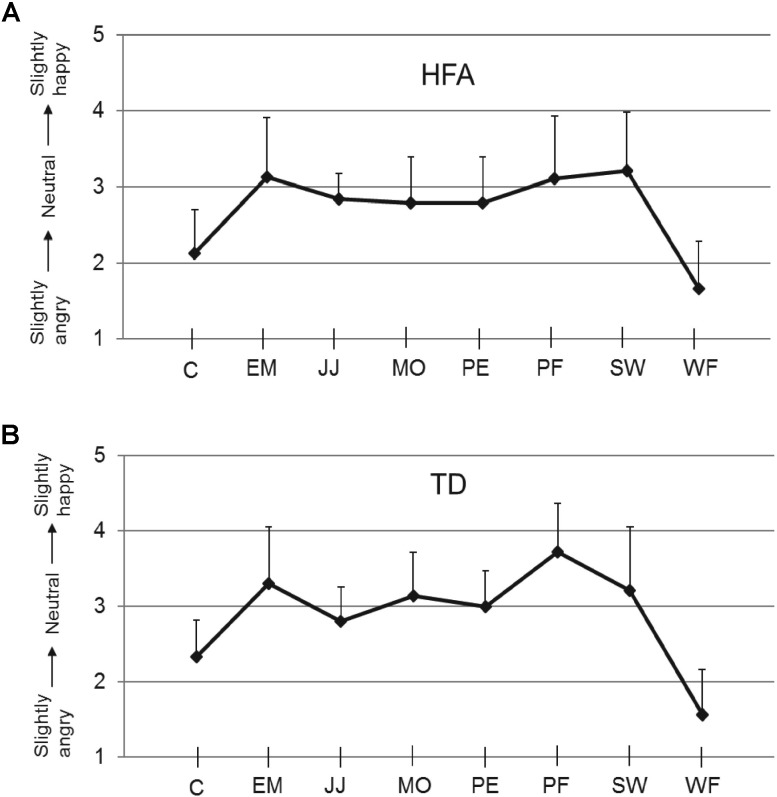
Calibration scores. Ratings on the 5-point scale (*y*-axis) for the neutral expressions of each of the eight actors used (*x*-axis), for HFA **(A)** and TD individuals **(B)**. Error bars indicate SEM. An illustration of the actors’ faces is in Palumbo and Jellema (2013).

The mean calibration scores for the neutral expression for each of the eight stimulus actors, obtained at the start of the experiment of each TD and HFA participant, are shown in **Figure 2**. The TD and HFA groups reported very similar scores, with the neutral expression of actors C and WF consistently rated as slightly angry. These calibration scores were used to adjust the scores in the subsequent experimental trials on an individual participant basis for each actor: a calibration factor (equal to 3.00 minus the calibration score) was added to the experimental scores. All statistical analyses were performed on the calibrated scores. The finding that the HFA and TD groups produced very similar evaluations of the “neutral” expressions of the eight actors, and in particular that all individuals of both groups consistently rated actors C and WF as slightly angry (**Figure 2**), indicates that HFA individuals did not show anomalies in processing subtle differences in these facial expressions. These results also mirror those in our previous studies (Palumbo et al., 2015).

### Data Reduction and Analysis

Trials in which RTs were below 250 ms or above 3000 ms were considered outliers and were removed (HFA, 10.4%; TD, 3.5%). Participants were excluded if more than 25% of their RT values fell outside the above range (HFA, *n* = 2; TD, *n* = 0) and when they pressed the same key for more than 90% of trials (HFA, *n* = 0; TD, *n* = 2). A ± 2.5 SD rule was applied to the mean difference of the ratings per participant, i.e., rating in the Anger-to-neutral condition minus rating in the Joy-to-neutral condition (HFA, *n* = 1; TD, *n* = 2).

**FIGURE 3:**
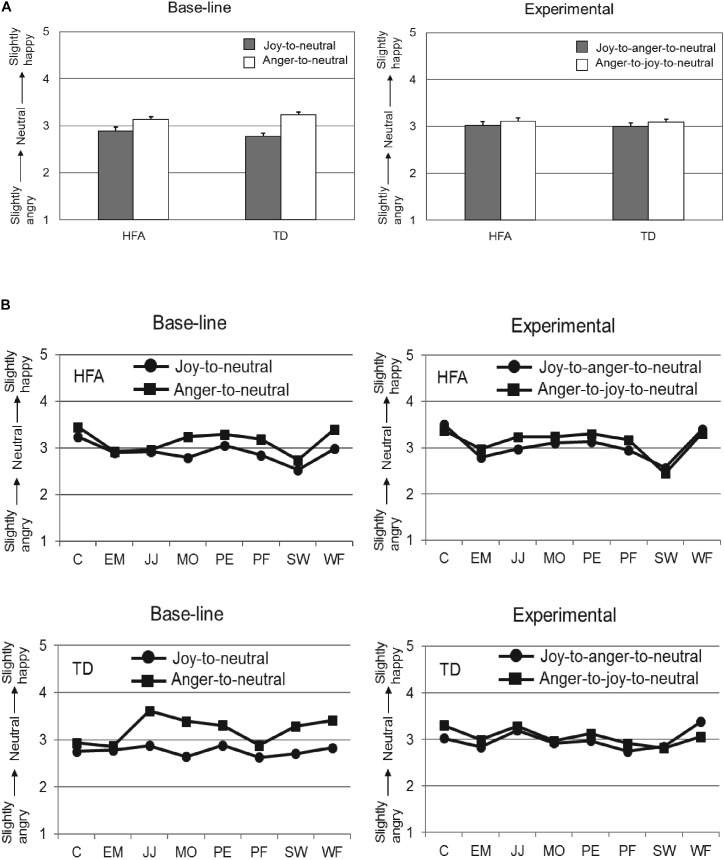
Mean ratings on the 5-point scale (*y*-axis) for the neutral expressions at the end of the video-sequences for the HFA and TD groups (*x*-axis) in the Offset condition **(A)** and in the Offset-onset-offset condition **(B)**. Error bars indicate SEM. Significant results are indicated with ^∗∗∗^*p* < 0.001.

**FIGURE A1:**
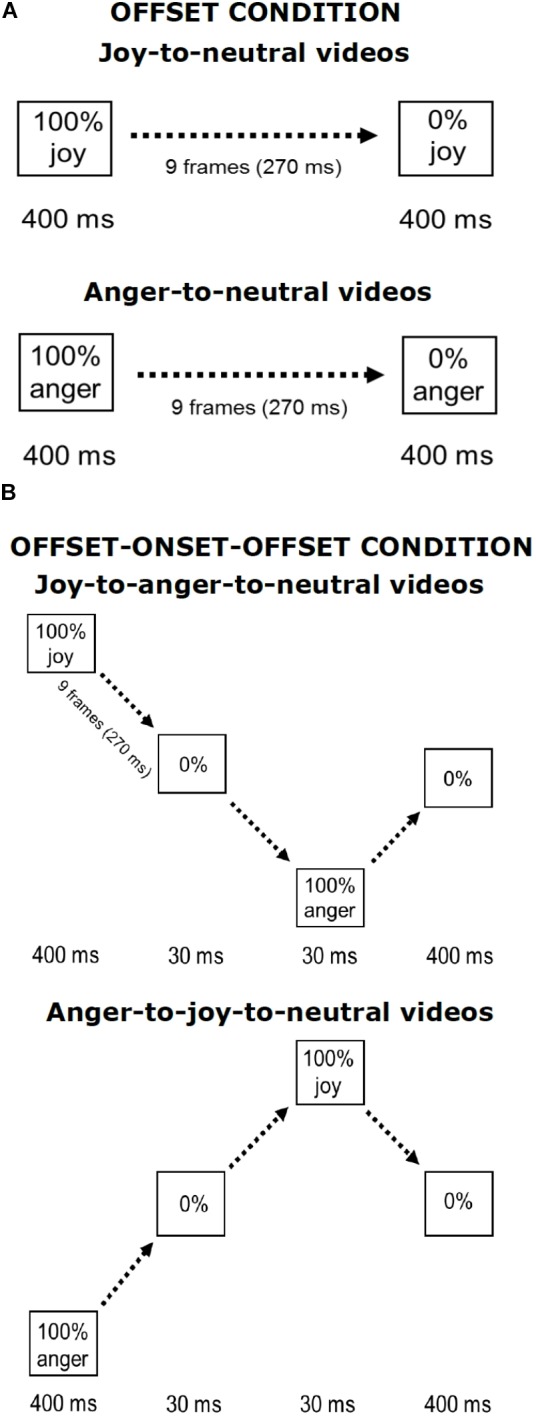
Mean ratings on the 5-point scale (*y*-axis) for the neutral expressions at the end of the video sequences for each of the eight actors (*x*-axis) in the Offset condition (left panels) and in the Offset-onset-offset condition (right panels) for the HFA (top row) and TD (bottom row) groups.

Following application of these exclusion criteria, the data of 18 TD individuals and 18 HFA individuals was analyzed with a 2 × 2 × 2 repeated measures ANOVA, with Offset history (Offset vs. Offset-onset-offset) and Initial emotion (Joy vs. Anger) as within-subject factors, and Group (HFA vs. TD) as between-subjects factor. The main effects of Offset history [*F*(1,34) = 2.14, *p* = 0.15, $\eta_{p}^{2}$ = 0.06] and Group [*F*(1,34) = 0.04, *p* = 0.85, $\eta_{p}^{2}$ = 0.00] were not significant, while the main effect of the factor Initial emotion was highly significant [*F*(1,34) = 40.44, *p* < 0.001, $\eta_{p}^{2}$ = 0.54], reflecting that the evaluations of the final neutral expressions were significantly different when the initial emotion was anger as compared to joy. Importantly, the interaction of Offset history by Initial Emotion was significant [*F*(1,34) = 6.96, *p* = 0.01, $\eta_{p}^{2}$ = 0.17]. *Post hoc* analyses showed the overshoot bias to be more pronounced in the Offset condition (Joy-to-neutral: *M* = 2.83, SD = 0.05; Anger-to-neutral: *M* = 3.19, SD = 0.04; paired samples *t*-test: *t*(35) = −5.97, *p* < 0.001) than in the Offset-onset-offset condition (Joy-to-anger-to-neutral: *M* = 3.01, SD = 0.06; Anger-to-joy-to-neutral: *M* = 3.10, SD = 0.05; paired samples *t*-test: *t*(35) = −1.31, *p* = 0.20). The interactions Offset history by Group [*F*(1,34) = 0.05, *p* = 0.82, $\eta_{p}^{2}$ = 0.00] and Initial emotion by Group [*F*(1,34) = 2.26, *p* = 0.14, $\eta_{p}^{2}$ = 0.06] were not significant, nor was the 3-way interaction [*F*(1,34) = 1.12, *p* = 0.30, $\eta_{p}^{2}$ = 0.03]. Thus, the TD and HFA groups responded in a very similar fashion in both conditions, with a significant overshoot response bias in the Offset condition and an absence of a response bias in the Offset-onset-offset condition. The results are shown in **Figure 3** (to illustrate consistency in these effects across the eight different actors, group means separated per actor can be found in **Figure A1**).

As in the calibration phase the “neutral” expressions of actors C and WF were consistently evaluated as slightly angry, whereas the other six actors were consistently evaluated as fairly neutral (see **Figure 2**), we conducted the same analyses on the data from just these six actors (i.e., excluding C and WF). This, however, resulted in the same outcome as was obtained for all eight actors.

## Discussion

The current study examined whether pattern extrapolation might give rise to distortions in the perception of dynamic facial expressions in individuals with HFA. Pattern extrapolation refers to the human tendency to detect patterns in presented stimuli and to extrapolate them. We argued that an “overshoot” response in the new experimental condition (happy-to-angry-to-neutral and angry-to-happy-to neutral) would support the notion that pattern extrapolation could underpin perceptual distortion in the HFA. Individuals with HFA are adept at detecting regularities and cause-effect relations, which typically rule object dynamics. Importantly, they may apply this vision of a rigid, rule based, environment to the social world to make sense of social signals (Vivanti et al., 2011; Hudson et al., 2012).

We found that in the Offset condition, offsets of happy and angry facial expressions reproduced the robust overshoot bias that was first reported in Palumbo and Jellema (2013): the last neutral expressions of the Joy-to-neutral and Anger-to-neutral videos were misjudged as slightly angry and slightly happy, respectively, in both groups. However, in the Offset-onset-offset condition we found an absence of the perceptual bias in both HFA and TD groups. These latter results suggest that pattern extrapolation did not play a major role in bringing about the overshoot bias in the Offset condition in the HFA group. Extrapolation of the facial expression dynamics would have resulted in a slightly happy evaluation of the neutral expression at the end of the joy-to-anger-to-neutral clips, and in a slightly angry evaluation of the neutral expression at the end of the anger-to-joy-to-neutral clips, whereas the results showed no response biases.

We previously suggested that the most likely mechanism underpinning the overshoot bias in the Offset condition in HFA was a sequential contrast effect, as the HFA group continued to show the perceptual bias after the agent’s identity had changed at the end of the clip (Palumbo and Jellema, 2013; Palumbo et al., 2015). Although the change of the agent’s identity suggested that the HFA group could have relied on sequential contrast effects, in these previous studies pattern extrapolation could not be excluded as explanatory mechanism. The results of the current experiment make this explanation very unlikely, as we found no evidence for an extrapolation of the observed pattern in the HFA group in the Offset-onset-offset condition. Therefore, the original suggestion that sequential contrast effects are the best candidate for explaining the overshoot bias in the HFA group still stands. This interpretation is also supported by the “Loop” condition (neutral-to-happy-to-neutral, and neutral-to-angry-to-neutral; Palumbo et al., 2015), where the contrast hypothesis would predict the absence of a perceptual bias (because the contrast is between the first and last frames, each presented for 400 ms, which were both neutral), which was exactly what was found. However, it should be stressed that the results from the current study on themselves do not allow to make any inference about the specific mechanism that underpinned the response bias in the HFA group. It merely allows to conclude that it was not pattern extrapolation or RM that caused the bias.

We previously argued that for the TD group the perceptual bias could not be explained by contrast effects, as the agent’s identity-change does not interfere with the contrast between the first emotional expression and the last neutral frame, and no bias was reported by the TD individuals in the identity-change condition. We therefore proposed an emotional anticipation mechanism (i.e., a low-level mind reading mechanism; Goldman, 2006) for the TD individuals, which would be susceptible to top-down information, such as identity information. The emotional anticipation hypothesis would predict the absence of a response bias in the new manipulation presented in the current study, which is what we found. The rationale is that because the perceptual history is equally divided over the two “opposite” emotions (joy and anger), the final neutral expression adequately sums up the agent’s (final) emotional state of mind.

Thus, the current study ruled out an explanation of the overshoot bias based on pattern extrapolation in both HFA and TD, while the findings are compatible with the notion that the perceptual bias in the Offset condition was caused by sequential contrast effects in HFA and by emotional anticipation in TD, but does not itself provide any new evidence for the latter.

### Emotional Anticipation: An Implicit Mechanism of Social Understanding

On the basis of our previous studies (Palumbo and Jellema, 2013; Palumbo et al., 2015) in conjunction with the current study, we propose to extend the notion of “perceptual mentalizing” (Teufel et al., 2010) by suggesting that the perceptual processing of social actions also interacts with implicit attributions made on the basis of the immediate perceptual history (Palumbo, 2012). These latter attributions are thought to reflect the operations of an anticipation mechanism, which operates automatically and involuntarily, not involving any deliberate reasoning, and which could be considered part of the perceptual system (Palumbo, 2012). In Teufel et al. (2010) model the observer is fully aware of the attributions, as they reflect explicit knowledge provided by the experimenter to the observer prior to the task. In our model, the processing of the dynamic facial expressions generates, in an automatic/involuntary fashion, an anticipation in the observer about what the actor’s most likely next mental/emotional state of mind will be. This happens “on line” during the task, whereby the most likely next state of mind is continuously updated on the basis of the immediately preceding events (Palumbo, 2012). These ideas blur the distinction between perception and mentalizing, as the latter is embedded within the perceptual process. It is as if the mere perception of the social stimulus automatically induces “mentalizing” activities, which then in turn modulate the perception (cf. Hudson et al., 2009; Hudson and Jellema, 2011).

We postulate that in ASD there may be an impairment in the ability to generate anticipations about the other’s immediate future action, or future state of mind, on the basis of the immediately preceding perceptual history, which could explain at least part of the communication difficulties they experience during social interaction. Taken together it suggest that individuals with HFA use an alternative route, which may rely more on physical characteristic rather than social meaning. The proposed mechanism of emotional anticipation matches recent theories of embodiment of facial expressions, which proposed that the categorization of facial expressions could be determined, or facilitated, by the experiential understanding of the agent’s emotional/mental state (Wicker et al., 2003; Botvinick et al., 2005). As such, emotional anticipation fits in well with embodied simulation models (Gallese, 2007; Niedenthal et al., 2010), which emphasize that the recognition of facial expressions is not purely the result of visual processing, but also relies on motor simulation (Palumbo, 2012). Recently substantial evidence has accumulated that the observation of dynamic facial expressions activates mirror neuron mechanisms (Dapretto et al., 2006; Pitcher et al., 2008; Likowski et al., 2012). Mirror neuron mechanisms have been argued to provide the observer with a notion of the upcoming action before it is executed (Fogassi et al., 2005; Cattaneo et al., 2007). As such, mirror mechanisms may underpin emotional anticipation (Palumbo, 2012). However, at this stage, direct evidence for this interpretation is not yet available and future research should shed light on the possible contribution of a simulation account.

## Ethics Statement

The study was approved by the Ethics Committee of the University of Hull and was conducted in accordance with the Declaration of Helsinki (2008). All participants signed written informed consent before taking part.

## Author Contributions

LP co-designed the study, carried out data collection for the TD group, performed the statistical analyses, interpreted the data, and drafted the manuscript. SM carried out data collection for the HFA group and helped in revising the manuscript. TJ co-designed the study, supervised the data collection, analysis and interpretation, and helped in revising the manuscript. All authors read and approved the final manuscript.

## Conflict of Interest Statement

The authors declare that the research was conducted in the absence of any commercial or financial relationships that could be construed as a potential conflict of interest.
